# Glomus tumor of the foot dorsum: A case report of a neglect podiatric entity

**DOI:** 10.1002/ccr3.9021

**Published:** 2024-05-30

**Authors:** Muhammad Imran, Yehya Khlidj, Ahmed Jahanzeb, Dawood Azam Farooq, Ateeba Kamran, Nour Fakih, Muhammad Abbas

**Affiliations:** ^1^ University College of Medicine and Dentistry, The University of Lahore Lahore Pakistan; ^2^ Faculty of Medicine University of Algiers 1 Algiers Algeria; ^3^ Karachi Medical and Dental College Karachi Pakistan; ^4^ Department of Natural Sciences Lebanese American University Beirut Lebanon; ^5^ Department of Plastic Surgery University College of Medicine and Dentistry, The University of Lahore Lahore Pakistan

**Keywords:** case report, dorsum of the foot, excision surgery, glomus tumor, painful nodule

## Abstract

**Key Clinical Message:**

Podiatrists and orthopedists should be vigilant for chronically evolving, hyperalgic soft lumps in the foot with vascular radiological features, prompting early detection of glomus tumor, timely mass removal, providing pain relief and improving patient's quality of life.

**Abstract:**

Glomus tumors refers to a rare group of benign perivascular neoplasms that originate from a neuromyoarterial structure called a glomus body. These tumors are characterized by their painful nature and predominant distribution in the extremities mainly the fingers, the hands and the feet. Nonetheless, the diagnosis is usually made after several years of symptoms experience as the lesions are mostly small, not palpable, and have variable presentations. Radiological workup especially with magnetic resonance imaging is very useful for diagnosing the tumoral process, however, confirmation can only be obtained by histological analysis. The treatment is purely surgical, and it is successful in most cases. Herein, we describe a case of glomus tumor of the foot dorsal side among a middle age male patient.

## 1 INTRODUCTION

Glomus tumor (GT) is a rare benign vascular neoplasm arising from the glomus body. The latter is a structure responsible for regulation of the corporeal temperature and skin circulation.^1^ Masson was the first to describe GT in 1924 as he observed that the hyperplastic degeneration in this lesion contained pathological features reminiscent of the glomus body.^2^ The most commonly involved sites include the fingers' subungual region as well as the palm, wrist, forearm, and foot. GT usually presents as a solitary lesion but in some cases multiple lesions may co‐develop.^2^ In other cases, the lesion can be associated with different tumoral types such as neuroma.^3^

Due to its progressive evolution, patients experience symptoms for 7–11 years on average before the diagnosis is made.^4^ The most typical symptoms include the classic triad of gradually severe pain (present in 100% of the cases), pinpoint tenderness, and cold hypersensitivity.^5^ The painful nature of GT leads to impairment in the patients' quality of life (QoL), which triggers the patient to seek healthcare advice. Radiological investigations such as ultrasound (US) and magnetic resonance imaging (MRI) helps in detecting the lesion with greater sensitivity; however, ultimate diagnosis requires the histopathological analysis.^4^, ^6^ The management of GT relies mainly on surgical excision which provides effective pain relief and QoL improvement.^7^ In this paper, we present a case of an adult male patient who experienced chronic podiatric pain due to a progressively evolving solitary GT that developed over the fourth tarsometatarsal joint of the right foot. The lesion was successively managed with surgical removal.

## 2 CASE HISTORY/EXAMINATION

A 55‐year‐old male presented to the plastic surgery department with a 5‐year history of localized aching pain and swelling on the dorsum of his right foot. Over the course of 5 years, the pain and swelling had progressively worsened, significantly impacting his QoL, particularly his ability to sleep at night. The patient reported that the pain intensified upon exposure to cold temperatures and even with the lightest touch. Walking was impossible, the symptoms were not alleviated by medical treatment. Notably, there was no history of trauma, and the patient had no coexisting medical conditions. His past medical and surgical history was unremarkable.

**FIGURE 1:**
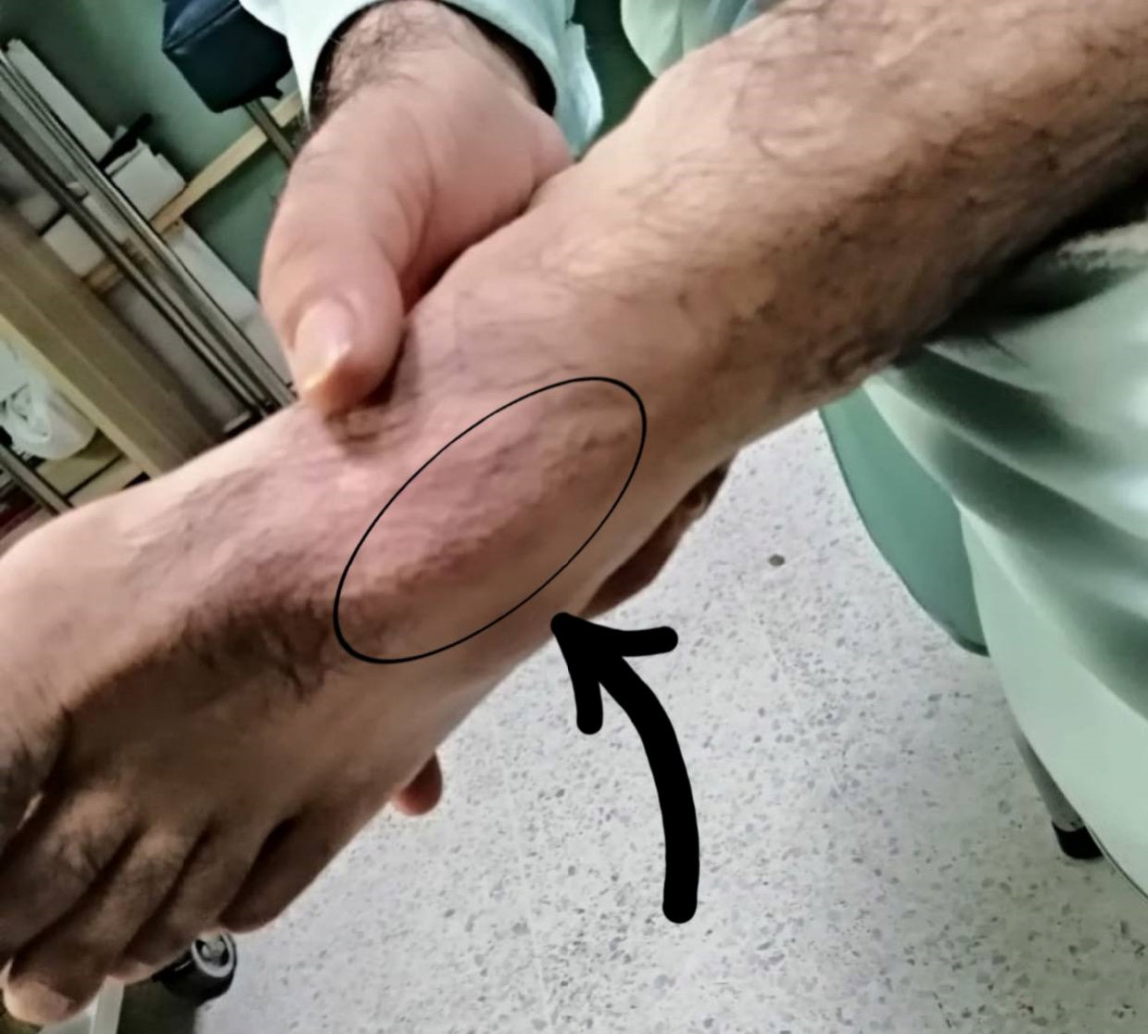
Preoperative image illustrating a 2 × 2 cm swelling located on the dorsum of the lateral aspect of the foot, specifically over the fourth metatarsal.

On physical examination, a small, soft, nodular swelling measuring approximately 2 × 2 cm was palpable on the dorsum of the foot (Figure 1). The tenderness upon touch was notably pronounced.

## 3 METHODS (DIFFERENTIAL DIAGNOSIS, INVESTIGATIONS, AND TREATMENT)

**FIGURE 2:**
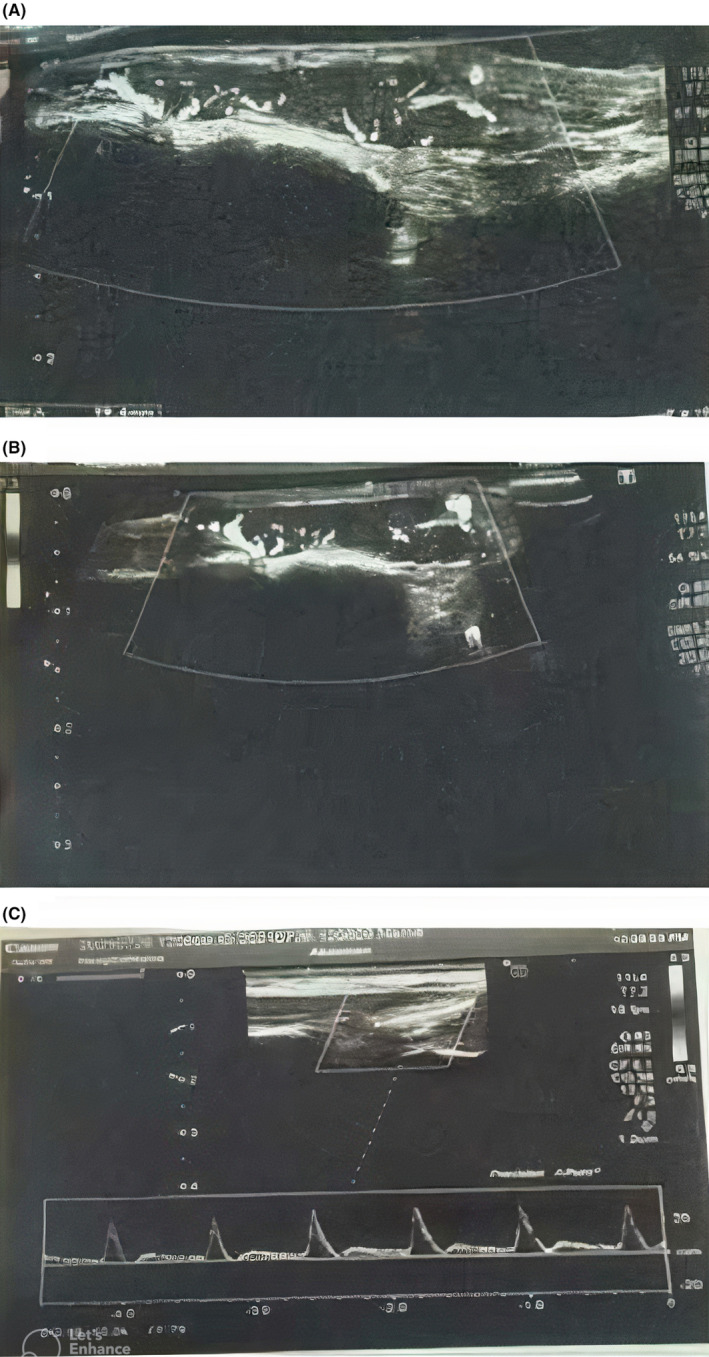
Lesion ultrasound findings showcase a 23 × 5 mm subdermal hypoechoic area over the fourth tarsometatarsal joint, featuring vascularity within and around the lesion, and involvement of the adjacent tendon, with no signs of liquefaction or calcification.

An ultrasound investigation revealed a linear subdermal hypoechoic area measuring 23 × 5 mm, directly overlaying the fourth tarsometatarsal joint at the location of the palpable lump. This lesion exhibited both internal and peripheral vascularity and appeared to encompass the adjacent tendon. Notably, no signs of internal liquefaction or calcification were observed (Figure 2). Based on these clinical and radiological findings, a preliminary diagnosis of GT was suspected, and surgical resection was planned. Preoperatively, patient all the baseline investigations were done and normal. The results are given below:

1. WBC count = 10.3 × 10^9^/L.
2. RBC count = 5.7 × 10^12^/L.
3. Hb (hemoglobin) = 11.2 g/dL.
4. Platelets count = 175 × 10^9^/L.
5. INR = 0.9.
6. PT/APTT = 11/29 s.
7. HbsAg = Negative.
8. Anti‐HCV = Negative.
9. Blood sugar random = 121 mg/dL.

**FIGURE 3:**
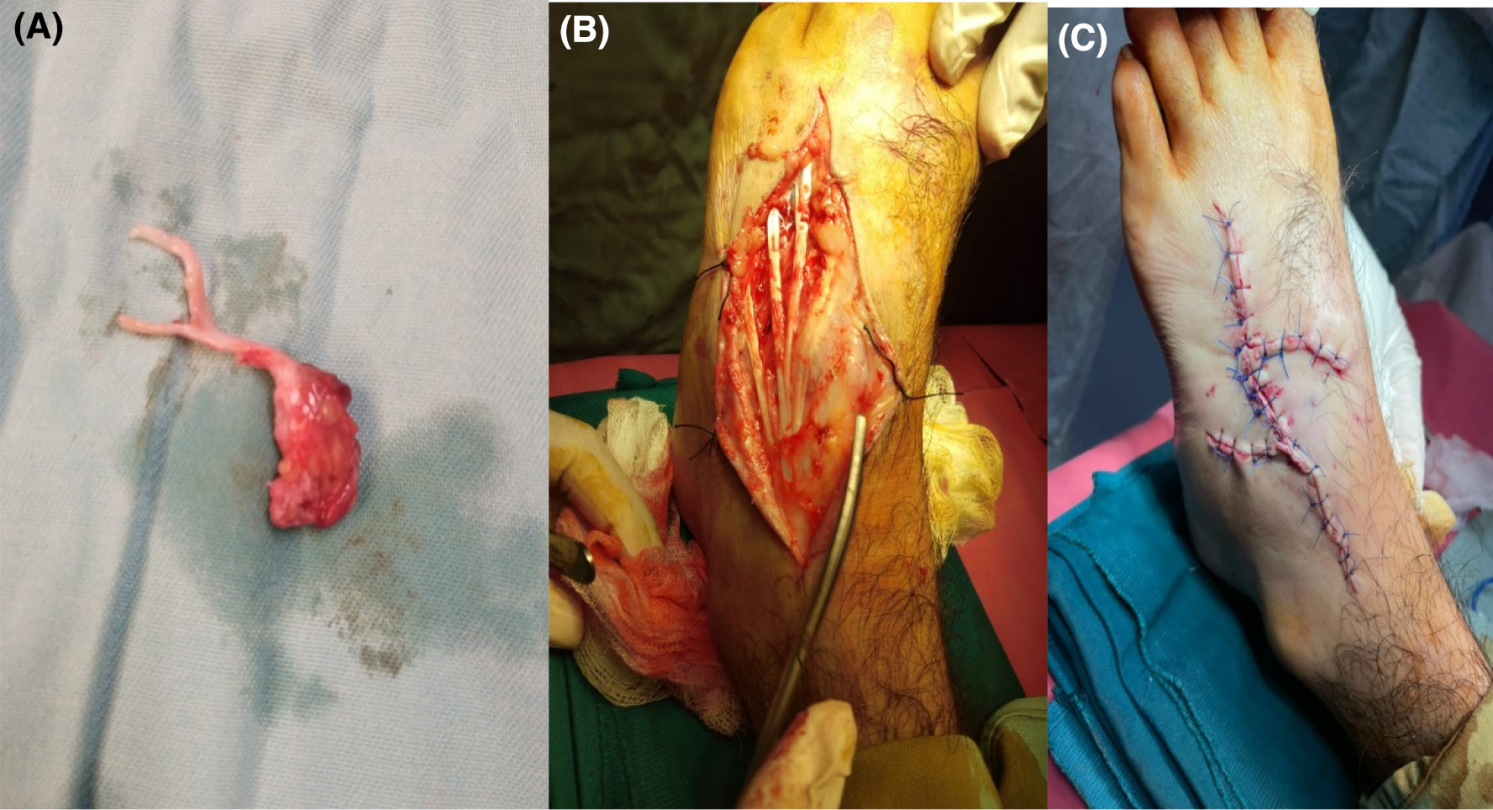
(A) Excised glomus tumor mass; (B) defect after excision of glomus tumor mass up to the deep fascia and overlying skin; (C) closure of defect with local transpositional flaps.

The surgical excision of the mass was performed under spinal anesthesia (Figure 3). The procedure was uneventful, with no intraoperative complications reported. Postoperatively, the patient was given analgesics and antibiotics. He experienced a smooth and uncomplicated postoperative recovery and was discharged on the third postoperative day.

## 4 CONCLUSION AND RESULTS

Histopathologic examination of the excised mass confirmed the diagnosis of a GT. Microscopically, the mass was composed of polygonal cells characterized by moderately distinct cellular borders and moderate cytoplasm with round nuclei. Thin‐walled blood vessels with bland endothelial cells were observed within the tumor. While tumoral cells were positioned immediately adjacent to the endothelial cells. No evident mitotic activity, focal nuclear enlargement, or nuclear atypia were identified, and tissue margins were clear.

The patient was scheduled for regular follow‐up visits. After approximately 2‐year follow‐up up till now, the patient reported being entirely pain‐free and had no recurrence.

## 5 DISCUSSION

This is of a 55‐year‐old male patient who developed an unusual GT of the right foot dorsum located at the subdermal area of the fourth tarsometatarsal joint. Due to the rarity of this type of neoplasms, their exact prevalence is unknown; however, it was that they account for less than 2% of soft tissue tumors.^8^ GT mostly predominates in the subungual regions of the fingers and the deep dermis of the palm, wrist, forearm, and foot as these sites contain high concentrations of glomus bodies.^9^ Glomus tumor are uncommon in the foot.^10^ Previously, Michal et al. presented a case of a 48‐year‐old man who complained of a painful mass in the dorsal side of the right foot that corresponded to a GT. Notably, the tumor showed signs of intravascular spread that resembled to that of intravascular leiomyomatosis without indicating any malignant character.^11^ Valero et al. also reported a giant glomus neoplasm that was located in the dorsal‐distal zone of the fifth ray. Moreover, the tumor was associated with a neuroma under the fifth metatarsal head.^3^ Moreover, Trehan et al. published a series of 11 podiatric GT cases highlighting their clinical, radiology, and pathology features as well as their surgical outcomes. In this series, the toe was most common site of tumoral lesions, the mean age of diagnosis was 45.4 years and MRI was the most effective diagnostic tool.^12^

The age of our patient is typical for GT as the diagnosis is mostly made in the fourth to seventh decades of life.^6^ The patient's sex is also consistent with the extradigital localization as the latter is more common among males, while the subungual lesions occur predominantly among females.^13^ The chief symptom was a gradually worsening pain which is likely more pronounced when GT arises from the foot due to higher susceptibility to repetitive mechanical trauma (e.g., from walking, running or long‐standing position) especially when wearing narrow shoes or high heels. A particular description of the pain was the fact that it was exacerbated by cold exposure (allodynia) and light touch (hyperalgesia) which demonstrates the hyperalgic characteristic of GT of the foot dorsum by possible compression/irritation of the cutaneous branches of the superficial peroneal nerve. In particular, pain, mild tenderness, and cold hypersensitivity were described in 80%, 100%, and 63%, respectively among GT patients.^14^ Our patient suffered from chronic localized podiatric pain during 5 years which is conform with the usual time before diagnosis due to the small size and slow growth rate of GT leading to delayed detection.

Radiological workup with US and MRI provides a useful diagnostic tool with a sensitivity reaching 82%–90%.^4^ Nevertheless, MRI had a distinct advantage in the diagnosis of glomus tumors, but its use is limited in developing countries for glomus tumor detection due to its high cost. With the improvement of resolution, high‐frequency US can not only clearly show the characteristics of the tumor in real time, but can also accurately locate the position, which is why it is more widely used in the preoperative examination of glomus tumors.^15^ Previous literature has shown the classical features on the doppler US of glomus tumor lesion as solitary hypoechoic lesions with clear boundaries and regular shape, and internal abundant flow signals,^16^ which is corresponding to our patient's lesion findings on US. The differential diagnoses of GT are represented essentially by other benign soft tissue neoplasms including lipoma, cyst, darkly colored masses (i.e., nevi, melanoma, angioma, or pyogenic granuloma) or those presenting as painful subcutaneous nodules (i.e., neuroma, leiomyoma, or spiradenoma).^6^

Ideally a biopsy should be performed before removing any neoplastic lesion that shows no obvious features of the relatively common benign podiatric tumors including mainly synovial cyst and plantar fibromatosis.^14^ However, in our case, the findings of clinical examination (notably the lesion’ tenderness and painful nature) along with the typical doppler US results (internal and peripheral vascularity with no liquefaction or calcifications) were suggestive of a benign tumor arising from vascular structure which imposed the consideration of the bleeding risk when sampling the lesion. The reason why excision biopsy is the most practiced simultaneously diagnostic and therapeutic approach when suspecting a GT.^4^

GT significantly alerts the QoL and overall has no tendency to regress spontaneously, mini‐invasive surgical excision is the exclusive treatment.^7^ Over 90% of the glomus tumor are benign and hence, wide local excision is the preferred treatment of choice, incision and approach depends upon the location of the glomus tumor. Our patient had a glomus tumor on the foot dorsum, hence a local excision with flap covering was done. Majority of the cases are subungual, and two different incision techniques are adopted including trans‐ungual and lateral subperiosteal approach depending on whether the lesion located centrally or peripherally, respectively.^17^ The recurrences may occur even after 9 years from surgery which imposes a relatively regular and long‐term follow‐up.^18^

The diagnosis of GT is challenging due to both lesion rarity and slow growing nature. However, it should be suspected by podiatrists and orthopedists whenever facing a chronically evolving hyperalgic soft lump in the foot that displays radiological features of vascular origin. Notably, this would allow earlier detection and mass removal, with subsequent pain relief and QoL amelioration.^19^

## AUTHOR CONTRIBUTIONS

**Muhammad Imran:** Conceptualization; supervision; validation; visualization; writing – original draft; writing – review and editing. **Yehya Khlidj:** Writing – original draft; writing – review and editing. **Ahmed Jahanzeb:** Writing – original draft. **Dawood Azam Farooq:** Writing – original draft; writing – review and editing. **Ateeba Kamran:** Writing – original draft; writing – review and editing. **Nour Fakih:** Supervision; writing – original draft. **Muhammad Abbas:** Supervision; validation.

## CONFLICT OF INTEREST STATEMENT

None.

## FUNDING INFORMATION

None.

## ETHICS STATMENT

The publication of this case report has been authorized by the quality service of our institution because case reports are exempted from ethical approval in our institute.

## CONSENT

Written informed consent was obtained from the patient to publish this report in accordance with the journal's patient consent policy.

## Data Availability

Due to privacy and ethical restrictions, the data supporting the findings of this study are available from the corresponding author only upon reasonable request.
